# State Gun Laws and Firearm-Related Homicides and Suicides, 2017-2022

**DOI:** 10.1001/jamanetworkopen.2025.19955

**Published:** 2025-07-11

**Authors:** Emma Cornell, Bailey Roberts, Rafael Klein-Cloud, Colleen P. Nofi, Chethan Sathya

**Affiliations:** 1 Center for Gun Violence Prevention, Northwell Health, New Hyde Park, New York; 2 Division of Pediatric Surgery, Cohen Children’s Medical Center, New York; 3 Donald and Barbara Zucker School of Medicine at Hofstra/Northwell, Hempstead, New York; 4 Institute for Health System Science, Feinstein Institutes, Northwell Health, New York

## Abstract

This cross-sectional study investigates the correlation of state gun law strength with firearm-related homicides and suicides from 2017 to 2022.

## Introduction

Firearms resulted in 48 204 deaths in the US in 2022.^1^ Root causes for firearm injuries from homicide or suicide or unintentional injuries differ significantly, and thus the impact of public policies may vary. Previous studies showing that states with strong gun laws have fewer firearm-related deaths have largely focused on the association of cumulative gun law scores or select laws, such as Child Access Prevention laws, with overall firearm-related mortality.^2,3,4^ However, little data exist evaluating the nuanced association of different gun laws with varying intents of firearm death, such as suicide and homicide. For example, policies restricting access to firearms during crisis may be associated with reduced firearm suicide, while safe firearm storage policy may be associated with reduced unintentional injury and policies addressing social determinants of health may have a stronger association with homicide. We evaluated correlations between strength of gun law categories and homicide and suicide, controlling for sociodemographic factors. By stratifying the association of firearm policies with differing firearm intents, we sought to provide a nuanced understanding of potential outcomes associated with tailored policy interventions.

## Methods

This cross-sectional study is reported following the STROBE reporting guideline and was exempt from review board (IRB) approval per Northwell Health IRB policy because it used deidentified data. State gun law strength was obtained from the Giffords Law Center across 10 categories (eMethods in Supplement 1). Giffords Gun Law scores from 2017-2021 were correlated with crude firearm death rates (FDRs) derived from CDC WONDER. County demographic data were obtained from the US Census ACS 5-year survey. FDR by intent (homicide or suicide) for each state was compared with state Giffords law score.^5^ Univariable and multivariable linear regressions were conducted by county level for homicide and suicide using combined data from Giffords, CDC, and ACS survey. *R*^2^ values representing strength of correlation were reported, with stronger correlations having *R*^2^ values greater than 0.10.^6^ Associations between gun law strength and FDR were analyzed by comparing the absolute value of the β (slope) in multivariable regressions. We used SPSS software version 29.0.0.0 241 (IBM), with the level of statistical significance set at a 1-sided *P* = .05.

## Results

Overall, gun law scores were low, with 52% of states achieving an F grade and 16% an A grade in 2022. Scores across states did not change significantly during the study period. Increasing cumulative state law strength was correlated with decreasing FDR (*R*^2^ = 0.19; *P* < .001) throughout all years. From 2018-2022, gun law scores more strongly correlated with reductions in suicide (*R*^2^ range, 0.703 for 2018 to 0.649 for 2022; both *P* < .001) vs homicide (*R*^2^ range, 0.144; *P* = .01 for 2018 to 0.099; *P* = .04 for 2022). Although univariate analyses demonstrated negative correlations between many gun laws and FDR across intents, analyses revealed stronger positive correlations of sociodemographic variables, such as increasing Black population, poverty, and unemployment, with homicide (Table 1). After controlling for sociodemographic factors, law categories with the strongest statistically significant negative correlation with suicides were “Regulation of Sales and Transfers,” “Firearms in Public Places,” “Gun Owner Accountability,” and “Classes of Weapons.” The strongest negative correlation between gun law strength and homicide was for laws relating to “Consumer and Child Safety” and “Investigating Gun Crimes,” with overall weaker correlations among all laws and homicides compared with suicides (Table 2).

**Table 1.** Univariate Correlation of Strength of State Laws With Mortalities by State in 2022

Factor^c^	Overall firearm mortality^a^^,^^b^				Suicide^a^^,^^b^				Homicide^a^^,^^b^			
	*R* ^2^	*P* value	β (slope)	*T*	*R* ^2^	*P* value	β (slope)	*T*	*R* ^2^	*P* value	β (slope)	*T*
Demographics												
Race and ethnicity^d^												
Asian	0.14	<.001	−0.37	−13.78	0.23	<.001	−0.48	−18.59	0.08	<.001	−0.28	−6.38
Black	0.27	<.001	0.52	20.82	0.04	<.001	−0.21	−7.44	0.65	<.001	0.81	30.13
Hispanic	0.03	<.001	−0.16	−5.61	0.04	<.001	−0.19	−6.56	0.07	<.001	−0.26	−5.93
White	0.12	<.001	−0.35	−12.90	0.11	<.001	0.33	12.07	0.39	<.001	−0.62	−17.63
Median age, per 1-y increase	0.01	<.001	0.1	3.43	0.14	<.001	0.37	13.82	0.01	.02	−0.10	−2.32
Male	0.02	<.001	−0.13	−4.59	0.03	<.001	0.17	6.04	0.10	<.001	−0.32	−7.46
Socioeconomic factors												
Poverty^e^	0.35	<.001	0.59	25.22	0.03	<.001	0.18	6.23	0.50	<.001	0.71	21.91
High school graduate or higher	0.16	<.001	−0.40	−14.86	0.01	<.001	−0.12	<0.001	0.17	<.001	−0.41	−9.90
Median annual income, per $1 increase	0.37	<.001	−0.61	−26.24	0.22	<.001	−0.47	−18.12	0.31	<.001	−0.56	−14.88
Health insurance												
Private	0.36	<.001	−0.6	−25.71	0.14	<.001	−0.37	−13.60	0.30	<.001	−0.55	−14.53
Public	0.24	<.001	0.49	19.36	0.16	<.001	0.40	14.73	0.19	<.001	0.44	10.79
None	0.09	<.001	0.30	6.85	0.07	<.001	0.27	9.56	0.09	<.001	0.30	6.85
Unemployment rate	0.14	<.001	0.38	14.03	0	.98	0	−0.31	0.26	<.001	0.52	12.28
Disability	0.28	<.001	0.53	21.52	0.31	<.001	0.56	22.95	0.21	<.001	0.46	11.48
Giffords Law Strength score, per 1-unit increase												
Total	0.19	<.001	−0.44	−16.64	0.65	<.001	−0.81	−15.37	0.10	.04	−0.32	−7.93
Background checks and access to firearms	0.12	<.001	−0.35	−12.85	0.45	<.001	−0.67	−9.86	0.07	.046	−0.31	−8.40
Regulation of sales and transfers	0.15	<.001	−0.39	−14.45	0.66	<.001	−0.82	−15.35	0.02	.18	−0.21	−7.42
Gun owner accountability	0.22	<.001	−0.47	−18.07	0.54	<.001	−0.74	−14.33	0.06	.07	−0.28	−6.36
Firearms in public places	0.15	<.001	−0.39	−14.33	0.57	<.001	−0.76	−13.17	0.04	.096	−0.26	−7.17
Classes of weapons and ammunition/magazines	0.143	<.001	−0.38	−14.03	0.500	<.001	−0.71	−14.58	0.07	.08	−0.27	−4.21
Consumer and child safety	0.16	<.001	−0.40	−15.12	0.46	<.001	−0.67	−11.77	0.12	.02	−0.34	−7.15
Investigating gun crimes	0.13	<.001	−0.36	−13.11	0.34	<.001	−0.59	−12.11	0.12	.02	−0.35	−5.90
Local authority to regulate	0.08	<.001	−0.28	−9.80	0.25	<.001	−0.50	−8.21	0.03	.28	−0.17	−5.999
Community violence intervention initiatives	0.03	<.001	−0.16	−5.55	0.03	.22	−0.18	−6.45	0.02	.43	−0.13	−3.15

^a^ Correlations were made by county level. Giffords scorecard values from 2022 were used. All counties within 1 state were given the same scores for correlation analysis. Models were run separately for each law category due to strong collinearity between law strengths within each category. Variables included in the multivariable model included county demographics by race, age, and sex and socioeconomic variables by poverty, education, income, health insurance, and disability. Unintentional firearm deaths were excluded due to small absolute numbers.

^b^ *R*^2^, β, and *T* values were all rounded to the nearest 0.01 decimal place.

^c^ Factors were evaluated per 1–percentage point increase, unless otherwise noted.

^d^ Race was self-reported as obtained in the American Community Survey, with categories of American Indian or Alaskan Native, Asian, Black or African American, Hispanic or Latino, Middle Eastern or North African, Native Hawaiian or Pacific Islander, and White. Race and ethnicity were included because there is no shortage of evidence demonstrating that historical and ongoing social and economic inequality and structural inequities in the health care system manifest adverse health outcomes for minority racial and ethnic communities.

^e^ The American Community Survey uses a poverty threshold that uses a set of income thresholds that vary by family size and composition to determine who is considered to be living in poverty.

**Table 2.** Multivariable Analysis of Laws and Mortalities by Intent in 2022

Gifford law category	Overall firearm mortality		Suicide		Homicide	
	β (slope)	P value	β (slope)	P value	β (slope)	P value
Total	−0.282	<.001	−0.292	<.001	−0.144	<.001
Background checks and access to firearms	−0.143	<.001	−0.077	.006	−0.073	.07
Regulation of sales and transfers	−0.243	<.001	−0.261	<.001	−0.094	.005
Gun owner accountability	−0.275	<.001	−0.260	<.001	−0.108	<.001
Firearms in public places	−0.272	<.001	−0.280	<.001	−0.141	<.001
Classes of weapons and ammunition/magazines	−0.208	<.001	−0.230	<.001	−0.047	.13
Consumer and child safety	−0.203	<.001	−0.184	<.001	−0.099	.004
Investigating gun crimes	−0.151	<.001	−0.149	<.001	−0.064	.04
Local authority to regulate	−0.086	<.001	−0.112	<.001	−0.001	.97
Community violence intervention initiatives	0.004	.16	−0.007	.79	0.053	.10

## Discussion

In this cross-sectional study, stronger gun laws correlated with decreased overall firearm mortality, with the strongest correlations for decreased suicides. For firearm suicide, our analyses lend support to policies that regulate firearm sales, transfers, and permitting laws. While some gun law categories were correlated with decreased firearm homicide, sociodemographic factors, such as unemployment, poverty, and insurance status, correlated with larger changes, suggesting policies that address root causes of violence through economic mobility and access to robust social, health, and educational services may be associated with a greater reduction in homicides. Study limitations include that linear sociodemographic data were assessed at the county and not individual level, nonfatal firearm injuries were not included, enforcement of policies across states is variable, states with more counties have stronger influence in our model, outcomes related to policy implementation often require longer-term analyses, and several relevant interpersonal factors were not captured. This study reveals the differential association of gun laws with firearm suicide vs homicide, highlighting the need for tailored public health interventions depending on the prevalence of firearm injury intents within a community, rather than a one-size-fits-all approach. Future prospective longitudinal studies are needed to evaluate these policies at an individual outcome level, examine policy implementation at the state level, better understand outcomes associated with community violence intervention work, and evaluate correlations with unintentional and nonfatal firearm injuries.

## Notes

Supplement 1.
eMethods.

eReferences.

Supplement 2.
Data Sharing Statement
