# Supplementary material for: State Gun Laws and Firearm-Related Homicides and Suicides, 2017-2022
Source: JAMA Netw Open. 2025 Jul 11;8(7):e2519955. doi: 10.1001/jamanetworkopen.2025.19955 (PMC12254883; doi:10.1001/jamanetworkopen.2025.19955)
Supplement: Supplement 1. — eMethods. eReferences. [file jamanetwopen-e2519955-s001.pdf]

# Supplemental Online Content

Cornell E, Roberts B, Klein-Cloud R, Nofi CP, Sathya C. State gun laws and firearm-related homicides and suicides. *JAMA Netw Open*. 2025;8(7):e2519955. doi:10.1001/jamanetworkopen.2025.19955

**eMethods.**

**eReferences.**

This supplemental material has been provided by the authors to give readers additional information about their work.

© 2025 Cornell E et al. *JAMA Network Open*.

**eMethods**

*Data Sources*

Gun law strength was obtained from the Giffords Law Center to Prevent Gun Violence "Gun Law Scorecard" which assigns points for the strength of firearm laws across 10 categories: "Background Checks and Access to Firearms," "Regulation of Sales and Transfers," "Gun Owner Accountability," "Firearms in Public Places," "Classes of Weapons and Ammunition/Magazines," "Consumer and Child Safety," "Investigating Gun Crimes," "State Authority to Regulate," "Community Violence Intervention Initiatives," and "Other" [1]. This scorecard is updated annually accounting for changes in individual state legislation over time and enabling quantitative analysis of gun policy among all 50 states. For state-level analysis, Giffords scorecard values for each year from 2017-2022 were used. For county-level analysis, Giffords scorecard values from 2022 were used. For statistical models, all counties within one state were given the same scores for correlation analysis. Giffords assigns a "grade" (A-F) based on the total points for that years' laws. State letter grades were compared by year.

Data on firearm deaths were obtained from CDC WONDER. Crude firearm death rate, (FDR) defined as firearm deaths per 100,000 people, was used for all analyses. For state level analysis, FDR was correlated with Giffords score by year [2]. For county-level analysis, crude FDR over a 5-year period from 2017-2022 was used. In counties with absolute mortalities less than 20, data is considered unreliable by the CDC, and were excluded from correlation analyses. Demographic data were obtained from the U.S. Census American Community Survey (ACS) 5-year survey (2017-2021).

*Analyses*

© 2025 Cornell E et al. *JAMA Network Open*.

Total Giffords law scores were calculated by state. Crude FDR or rate by intent (homicide, suicide) for each state was compared to Giffords law score. Linear regressions were used to evaluate the relationship between law strength and FDR by year.

Univariable and multivariable linear regressions were conducted by county level using combined data from the Giffords scorecard, CDC WONDER and ACS survey. $R^2$ values representing strength of correlation were reported, with stronger correlations having $R^2$ values > 0.10 [3]. For multivariable regressions, models were run separately for each law category due to strong collinearity between law strengths within each category. Variables included in the multivariable model were county racial and ethnic demographics (percentages of White, Black, Asian, and Hispanic), median age, percent male, and socioeconomic variables including percent in poverty, high school graduation or higher, private, public, or no health insurance, disability, median income, and unemployment rate. Stratified univariable and multivariable analyses by firearm homicide and suicide were also performed. Associations between gun law strength and FDR were analyzed by comparing the absolute value of the beta (slope) in the multivariable linear regressions.

© 2025 Cornell E et al. *JAMA Network Open*.

eReferences

1. Violence, G.L.C.t.P.G. *Annual Gun Law Scorecard*. [cited 2024]; Available from: https://giffords.org/lawcenter/resources/scorecard/.
2. Centers for Disease Control and Prevention, N.C.f.H.S., CDC WONDER Online Database, *Provisional Mortality Data* in *National Vital Statistics System* 2024.
3. Ozili, P.K., *The acceptable R-square in empirical modelling for social science research*, in *Social research methodology and publishing results: A guide to non-native english speakers*. 2023, IGI global. p. 134-143.

© 2025 Cornell E et al. *JAMA Network Open*.
