# Supplementary material for: State Gun Laws and Firearm-Related Homicides and Suicides, 2017-2022
Source: JAMA Netw Open. 2025 Jul 11;8(7):e2519955. doi: 10.1001/jamanetworkopen.2025.19955 (PMC12254883; doi:10.1001/jamanetworkopen.2025.19955)
Supplement: Supplement 2. — Data Sharing Statement [file jamanetwopen-e2519955-s002.pdf]

Data Sharing Statement

Cornell. State Gun Laws and Firearm-Related Homicides and Suicides. *JAMA Netw Open*. Published online July 11, 2025. doi:10.1001/jamanetworkopen.2025.19955

Data
Data available: No

Additional Information
Explanation for why data not available: data is publicly available, however some of the Giffords data and specific scores cannot be made public as per our agreement with Giffords
